# Total laryngectomy and readmission: causes, rates and predictors

**DOI:** 10.1186/s13104-023-06645-z

**Published:** 2023-12-20

**Authors:** Almoaidbellah Rammal, Abdulsalam Alqutub, Omar Alsulami, Naif Mozahim, Sara Mozahim, Mohammed Awadh, Muatasaim Hakami, Rahaf AlThomali, Ahmed Mogharbel

**Affiliations:** 1 Otolaryngology–Head and Neck Surgery Department, King Abdulaziz University Hospital, King Abdulaziz University, Jeddah, Saudi Arabia; 2 Otolaryngology–Head and Neck Surgery Department, Faculty of Medicine, King Abdulaziz University, Jeddah, Saudi Arabia; 3 Otolaryngology–Head and Neck Surgery Department, King Fahd Armed Forces Hospital, Jeddah, Saudi Arabia

**Keywords:** Laryngectomy, Patient readmission, Surgical wound Infection, Retrospective studies, Hypoalbuminemia, Ethnicity

## Abstract

**Background:**

Total laryngectomy (TL) is a complex procedure, and patients undergoing TL are at high risk for readmission, which exposes them to hospital-acquired complications. Readmission rate is a metric for quality of care. We aimed to identify the rate, causes, and predictors of hospital readmission within 60 days after discharge following TL.

**Methods:**

This is a 12-year retrospective study where we included all patients undergoing TL in a single tertiary care center between 2008 and 2022. Patient charts were reviewed for demographics, comorbidities, and causes for readmission.

**Results:**

Of 83 patients who underwent TL, 12 (14.50%) were readmitted within 60 days. Common causes were surgical site infection (33.33%) and mucocutaneous fistula (25%). Significant predictors for readmission were tobacco use (P = 0.003), African ethnicity (P = 0.004), being unmarried (P < 0.001), lower preoperative serum albumin (P < 0.001), higher preoperative TSH (P = 0.03), higher preoperative neutrophil count (P = 0.035), higher American Society of Anesthesiology (ASA) score (P = 0.028), and higher Cumulative Illness Rating Scale (CIRS) score (P = 0.029).

**Conclusion:**

One in every seven patients were readmitted following TL. Frequent causes include wound infection and fistulas. Predictors include preoperative hypoalbuminemia, hypothyroidism, African ethnicity, being unmarried, tobacco use, and a higher baseline burden of comorbidities. Such factors can be targeted to reduce hospital readmission rates.

## Introduction

Total laryngectomy (TL) is a widely performed surgical procedure for treating laryngeal cancer [1]. While organ preserving management of laryngeal cancer is the preferred option of treatment, TL is still indicated in patients with advanced laryngeal malignancies, a failed response to chemotherapy or radiotherapy, and histopathological subtypes, which are known to be radiotherapy resistant [2–4]. Although TL is a relatively safe procedure, it still has a fair share of surgical complications. These complications include bleeding, airway compromise, fistulas, pharyngoesophageal stenosis, stoma stenosis, and hypothyroidism [5]. Due to these complications, patients who undergo TL are always at risk of readmission, which causes a burden to the patient and the healthcare system.

A single institution retrospective study conducted at an academic hospital found that patients who underwent TL were 4.7 times more likely to be admitted than other head and neck surgeries [6]. Published works regarding the rate of readmissions after laryngectomy ranged from 10.9 to 20.6%, with postoperative pharyngocutaneous fistula and postoperative infection as the most common cause of readmission [7–12]. Even with studies on the risk factors and problems that lead to readmission after laryngectomy, the rationale for the high readmission percentage in contrast to other head and neck surgeries remains unknown. Moreover, the rates and risk factors for readmission after laryngectomy need to be clearly described in the Saudi population. This study aims to determine the incidence, risk factors, and most likely complications that will cause readmission following laryngectomy within 60 days of hospital discharge at King Abdulaziz University Hospital in Jeddah, Saudi Arabia.

## Methods

After receiving ethical approval from the Institutional Review Board (IRB), we reviewed the records of patients who underwent TL between 2008 and 2022.

The causes of readmission were extracted as the final diagnosis from the medical record system. Only the first episode of unplanned returns was acquired if more than one episode was identified within the first 60 days after discharge.

We included all patients who underwent TL in our center between 2008 and 2022. Patients with significant missing data, such as not having any recorded causes for readmission were excluded from the study.

Demographic data such as age, gender and race, marital status, tobacco use, dates of first admission, discharge, and return were also gathered.

The records of the patients were also reviewed for any incidents that occurred before or during their initial hospitalization, such as any history of tracheostomy, intubation, or intensive care unit (ICU) admission. We also reviewed the records for any history of chemotherapy or radiotherapy.

Additionally, preoperative thyroid stimulating hormone (TSH), serum albumin, white blood cell count, platelets, and hemoglobin were collected.

The Cumulative Illness Rating Scale (CIRS) and the American Society of Anesthesiology (ASA) score were used to assess the patient’s comorbidities. The CIRS is a comorbidity scale that analyzes the disease burden across 13 body systems [13].

The data were entered into Google Forms and then exported to Excel 16.0. Statistical analysis was performed using the Statistical Package for the Social Sciences for Windows version 21.0 (IBM SPSS Statistics), with statistical significance set at P 0.05. Continuous variables are expressed as the means and standard deviations (SD) or median with interquartile ranges (IQR) depending on the distribution. Categorical variables are summarized using numbers and frequencies. Student’s t test was performed to compare means. The Mann‒Whitney U test was used to compare medians, while the chi-square test was used to compare frequencies. Variables with significant relationships in univariate analysis were employed in multivariate analysis.

## Results

Eighty-three cases met the study criteria, of which 12 (14.50%) were readmitted within 60 days after discharge. The mean time to hospital readmission was 26.66 ± 11.06 days (range 1–58 days). Table 1 shows the causes of readmission; the most common reason was surgical site infection (33.33%), followed by mucocutaneous fistula (25%).

**Table 1:** Rate and causes of readmission

Causes	Numbers	Rates (%)
Surgical site infection	4	33.33
Mucocutaneous fistula	3	25
Dysphagia	2	16.67
Equipment issues: tracheostomy, surgical drain	1	8.33
Stomal stenosis	1	8.33
Dyspnea	1	8.33

Significant predictors for unplanned readmission included tobacco use (P = 0.008), African ethnicity (P = 0.017), and being unmarried, separated, or widowed (P < 0.001). Lower preoperative serum albumin levels (10.5 vs. 23.83 g/L, P < 0.001), higher preoperative TSH levels (13.03 vs. 2.16 mIU/L, P = 0.031), and higher preoperative neutrophil count (5.87 vs. 4.01 K/µL, P = 0.037) showed significant associations with readmission within 60 days after discharge. Additionally, having a higher baseline burden of disease when using the ASA score (P < 0.001) and the CIRS score (P = 0.028) was shown to be a significant predictor for hospital readmission. A simple linear regression between the two scores revealed a significant positive correlation (p < 0.001), with a r^2^ = 0.409. Table 2 summarizes the characteristics of patients who underwent TL and compares readmitted and nonreadmitted patients after hospital discharge.

**Table 2:** Comparison between readmitted and nonreadmitted patients in gender, age, ethnicity, marital status, tobacco use, ASA score, and CIRS score, history of intubation, ICU admission, chemotherapy, radiotherapy, preoperative TSH, serum albumin, white blood cells, neutrophils, lymphocytes, and hemoglobin

Variable	Readmitted	Nonreadmitted	P value
Gender (n, %)			
Male	9 (75)	52 (73.20)	0.351
Female	3 (25)	19 (26.80)	
Age			
Mean (SD)	50.9 (18.55)	51.5 (6.74)	0.913
Ethnicity (n, %)			
Arab	1 (8.30)	25 (35.20)	0.017
Asian	3 (25)	29 (40.80)	
African	8 (66.70)	17 (23.90)	
Marital status (n, %)			
Married	2 (16.70)	69 (97.20)	< 0.001
Unmarried (single, divorced, separated)	10 (83.30)	2 (2.80)	
ASA			
Mean (SD)	3.75 (2.30)	2.3 (0.92)	< 0.001
CIRS			
Mean (SD)	9 (5.61)	5.58 (4.80)	0.028
Chemotherapy and/or radiotherapy (n, %)			
Chemotherapy	1 (8.33)	8 (11.30)	0.516
Radiotherapy	1 (8.33)	8 (11.30)	
Chemoradiotherapy	6 (50)	20 (28.20)	
Neither	4 (33.33)	35 (49.30)	
Tobacco use			
Yes	12 (100)	39 (54.90)	0.008
No	0 (0)	32 (45.10)	
ICU admission (n, %)			
Yes	9 (75)	52 (73.20)	0.351
No	3 (25)	19 (26.80)	
Intubation (n, %)			
Yes	8 (66.67)	52 (73.20)	0.351
No	4 (33.33)	19 (26.80)	
Preoperative TSH (mIU/L)			
Mean (SD)	13.03 (9.22)	2.16 (0.82)	0.031
Preoperative albumin (g/L)			
Mean (SD)	10.5 (7.88)	23.92 (9.15)	< 0.001
Preoperative white blood cell count (K/µL)			
Mean (SD)	9.19 (3.46)	7.6 (2.65)	0.068
Preoperative neutrophil count (K/µL)			
Mean (SD)	5.87 (4.03)	4.01 (2.52)	0.035
Preoperative platelet count (K/µL)			
Mean (SD)	351.833 (138.49)	295.92 (100.07)	0.095
Preoperative lymphocyte count (K/µL)			
Mean (SD)	1.84 (1.07)	2.05 (1.33)	0.612
Hemoglobin (g/dL)			
Mean (SD)	12.83 (1.49)	12.42 (1.83)	0.612

The multivariate logistic regression analysis revealed significant risk factors for readmission after hospital discharge, including history of tobacco use [odds ratio (OR) = 0.24; 95% confidence interval (CI) 0.08–0.39; P = 0.003], African ethnicity (OR = 0.14; 95% CI 0.05–0.23; P = 0.004), being unmarried, separated, or widowed (OR = 0.83; 95% CI 0.71–0.96; P < 0.001), lower preoperative serum albumin (OR = 0.51; 95% CI 0.34–0.67; P < 0.001), higher preoperative TSH (OR = 0.05; 95% CI 0.01–0.10; P = 0.03), higher preoperative neutrophil count (OR = 0.03; 95% CI 0.002–0.06; P = 0.035), higher ASA score (OR = 0.11; 95% CI 0.05–0.16; P = 0.028), and higher CIRS score (OR = 0.02; 95% CI 0.002–0.03; P = 0.029).

## Discussion

We present exclusive and distinct data about the causes of unexpected hospital readmission by extending the analysis period to 60 days rather than the traditional 30 days following surgery. Our study was conducted in a tertiary referral center in western Saudi Arabia; many cases are sent to our hospital from remote regions, and transportation and referral may interfere with early follow-ups. Thus, extending the study period to 60 days after discharge would enable us to understand better the actual rate of unexpected hospital readmission following TL.

In our study, the rate of unplanned hospital return after TL was 14.50%. This is higher than the readmission rates reported in other studies. Wu et al. reported a rate of 3.20% after head and neck surgeries [14]. Graboyes et al. identified a readmission rate of 7.30% for all otolaryngological procedures [11]. Conversely, Chaudhary et al. reported a rate of 14.10% after laryngeal and oropharyngeal cancer surgery [15]. Together with our study, these reports support that the readmission rates after laryngeal surgery are higher than those after other otolaryngological procedures. Our results of increased readmission rates following laryngeal cancer surgery may be attributable to the surgery’s greater complexity and the patients’ complexity compared to patients undergoing other otolaryngological procedures.

Although readmission rates have been employed as a quality metric for hospital care, their use has limitations. While there are potentially preventable reasons and risk factors for readmission, nonmodifiable factors can contribute to patient readmission, including socioeconomic status, race, age, and gender [16]. In our study, patients of African descent were more likely to be readmitted after hospital discharge. It has been reported that people of African ethnicity, rather than other races, are at increased risk of prolonged hospital stay, increased readmission rates, morbidity, and mortality [8, 17–19]. It is unclear why these racial disparities exist; however, previous literature suggests that African patients are more likely to present with advanced-stage disease, tobacco use, complicated comorbidities, and lack of access to healthcare, leading to poorer outcomes [8, 20]. All patient demographics must have adequate preoperative access to healthcare, yet traditionally underserved populations need special attention.

We found that a lower serum albumin level significantly predicts hospital return. Another study demonstrated that preoperative hypoalbuminemia is associated with higher morbidity, mortality, and postoperative complications, especially infections [21, 22]. Our study showed consistent results, as surgical site infection was the most common cause of hospital readmission after TL. In acute sickness and injury, serum albumin levels fall as the liver shifts the priority of protein synthesis from visceral proteins to acute-phase reactant proteins [23–25]. Thus, hypoalbuminemia may serve as a diagnostic tool for underlying systemic immunoinflammation.

Our study found that a higher preoperative TSH level is a risk factor for hospital readmission. Previous studies confirmed that operating on patients with overt or biochemical hypothyroidism is associated with adverse outcomes and prolonged hospital stays [26, 27].

Our study showed that patients who were unmarried, separated, or widowed were more prone to readmission. This finding aligns with other studies that revealed an association between social support and acute care needs. Wachtel et al. proved that spousal support, rather than any other family members, is a significant protective factor against unplanned hospital return after discharge [28]. Another study on patients undergoing laryngeal and oropharyngeal cancer surgery found that separation or divorce is an independent risk factor for hospital readmission [15]. These findings suggest that there is a high-risk population that should be targeted to produce interventions that prevent unplanned readmission after hospital discharge.

This study used two validated comorbidity scores to evaluate patients’ comorbidities and their predictive value for readmission after TL. Both scores were significant predictors of readmission within 60 days of discharge. Previous reports confirmed that the ASA score is closely linked to predicting readmissions and is positively associated with increased readmission rates [14, 29, 30]. Additionally, the CIRS comorbidity score has been previously used in head and neck cancer patients, with higher scores suggesting worsening baseline health [13, 14, 31]. It is well-recognized that patients with head and neck cancer have more comorbidities due to long-term exposure to risk factors such as tobacco use [32, 33]. More extensive and close postsurgery follow-ups for individuals with high baseline health burdens may reduce unplanned readmissions.

## Limitations

Our findings are to be interpreted with several limitations in mind. The usual concern for retrospective studies is obtaining reliable and conclusive data on the specific cause and time of hospital return postdischarge. For the same reason, it was challenging to collect other important variables such as the stage and type of laryngeal cancer, that may have impacted the results of our study. Other limitations were multiplicity of surgeons and variable expertise which are important predictors of outcomes. The fact that our study was limited to a single area may limit the generalizability of our findings. Further multicenter prospective research activities with extended follow-up periods in larger populations are thus desired.

## Conclusion

The rate of readmission after total laryngectomy was 14.50%. Common causes were wound infection and mucocutaneous fistula. Significant predictors include preoperative hypoalbuminemia, biochemical hypothyroidism, African ethnicity, being unmarried, tobacco use, and having a higher baseline burden of comorbidities. Such causes and risk factors can be targeted to reduce hospital readmission rates.

## Data Availability

Data were collected throughout the years 2022 and 2023 from the hospital’s Phoenix system and patients’ paper-based records and can be provided upon request for appropriate reasons.

## Abbreviations

TL: Total laryngectomy
ASA: American Society of Anesthesiology
CIRS: Cumulative Illness Rating Scale
TSH: Thyroid stimulating hormone
ICU: Intensive care unit
SD: Standard deviation
